# A systematic review and meta-analysis of epidemiology of depression in people living with HIV in east Africa

**DOI:** 10.1186/s12888-018-1835-3

**Published:** 2018-08-15

**Authors:** Getinet Ayano, Melat Solomon, Mebratu Abraha

**Affiliations:** 1 Research and Training Department, Amanuel Mental Specialized Hospital, Addis Ababa, Ethiopia; 2 Department of Psychiatry, Paulo’s millennium medical college, Addis Ababa, Ethiopia

**Keywords:** Prevalence, HIV, AIDS, Determinants, Factors, Depression, Systematic review, Meta-analysis, East Africa

## Abstract

**Background:**

Depression is the most prevalent psychiatric disorder among people living with HIV (PLWHIV) and is associated with poor quality of life, additional comorbidities, disability, unemployment, poorer therapeutic outcomes and risky behaviors. The present systematic review and meta-analysis aims to systematically summarize empirical evidence and to formulate recommendations for future research.

**Methods:**

We searched PubMed, EMBASE, SCOPUS, and relevant literature for possible studies to include. A qualitative and quantitative analysis was undertaken for this systematic review. Subgroup and sensitivity analysis were performed. Cochran's Q- and the I^2^ test were used to assess heterogeneity. The presence of publication bias was evaluated by using Egger's test and visual inspection of the symmetry in funnel plots.

**Results:**

Of 283 titles initially identified, 81 abstracts were eligible for review. Of these, 46 articles qualified for full text review and 19 were retained. In our meta-analysis the pooled prevalence of depression in PLWHIV was 38% (95% CI 29.30-47.54). The pooled prevalence estimates of depression was 49.79% in Ethiopia and 30.88% in Uganda. In addition, the prevalence of depression was 12.40% and 46% as measured by diagnostic and screening instrument respectively. Our qualitative synthesis showed that factors such as having opportunistic infection, perceived stigma, negative life event, WHO clinical staging of disease, hospitalization in the past one month, stressful life events, food insecurity, self-efficacy, missed frequency of clinic visit, frequency of follow-up, older age, low income, urban residence and being government employee were strongly and significantly associated with depression in PLWHIV in east Africa.

**Conclusion:**

The pooled prevalence estimates of prevalence of depression in PLWHIV was 38%. The prevalence estimates of depression in PLWHIV in Ethiopia was significantly higher than Uganda. In addition the prevalence of depression was significantly higher in studies conducted by screening than diagnostic instrument. Routine screening and integrated management of depression into the existing HIV care services is warranted. Validation and use of standard instrument to assess depression in PLWHIV is needed. Moreover, longitudinal and community based studies focusing on incidence and determinates of depression in PLWHIV are recommended.

**Electronic supplementary material:**

The online version of this article (10.1186/s12888-018-1835-3) contains supplementary material, which is available to authorized users.

## Background

Depression is the most prevalent psychiatric disorder among people living with HIV (PLWHIV) and is associated with poorer therapeutic outcomes and risky behaviors. Different epidemiological studies have estimated the prevalence of depression in PLWHIVto be between 2.27% and 76.7% [1–20]. In fact, it is one of the leading causes of morbidity and mortality among PLWHIV [21].

In the past decades, studies have determined the prevalence of depression in PLWHIV. However, the overall prevalence rate of depression in PLWHIV remains unclear in east Africa; previous studies reported prevalence rates between 2.7% and 76.7% [1–20]. Several factors may contribute to this wide range of prevalence rates, including: (i) differences between included PLWHIV with respect to the stages of HIV disease, treatment status and CD4 level (ii) the use of different instruments to assess depression with different psychometric properties; and (iii) the use of different criteria to define depression [22].

Depression is the most commonly experienced psychological conditions experienced by PLWHIV and is associated with poor quality of life [23], additional comorbidities [24], disability, and unemployment [25–27]. In addition, depression in PLWHV leads poor social conditions, poor treatment adherence and, poor therapeutic outcomes, and risky behaviors [28–31].

A number of factors can predispose PLWHIV to depression including, having opportunistic infection [9, 10], perceived stigma [2], negative life event [26], having severe WHO clinical stage diseases [2, 9, 10], stressful life events [26], food insecurity [9], older age [17], low income [3] and advanced HIV disease [22].

Evidence shows that east Africa is the region most affected by HIV in the world next to south Africa [32]. However, the there are no systematic review and meta-analysis studies conducted on the epidemiology of depression in PLWHIV in east Africa. Therefore, the main objective of this systematic review and meta-analysis is to summarize the available empirical evidence in east Africa on: [1] the prevalence of depression in people living with HIV (PLWHIV) and [2] the determinants of depressionin PLWHIV and to formulate recommendations for future clinical practice and research.

## Methods/design

We conducted extensive search of literature as indicated in the guideline of reporting systematic review and meta-analysis (PRISMA) [32]. Three databases such as PubMed, Embase, and Scopus were consulted for our literature search. We conducted our search in Pubmed using the following terms and keywords: "Epidemiology OR prevalence OR magnitude OR incidence AND depression OR depressive symptoms OR depressive disorder OR depressive AND HIV OR human immunodeficiency virus OR AIDS AND factor OR risk OR risk factor OR determinant AND Ethiopia OR Eritrea OR Kenya OR Uganda OR Tanzania OR Sudan OR Djibouti OR Somalia OR Rwanda OR east Africa". For the other two databases (Embase and Scopus) we employed specific-subjects headings as advised for each databases. In addition, in order to identify other relevant articles we manually searched the reference lists of eligible articles.

### Eligibility criteria

Two reviewers (GA and MA) evaluated the relevant articles using their title and abstracts prior to retrieval of full-text articles. The retrieved full-text articles were further screened according to pre-specified inclusion and exclusion criteria. We resolved disagreements by discussing with a third reviewer (MS).

### Inclusion criteria

- Design type -Cross sectional and other observational studies
- Study subject– people living with HIV
- Articles published in English language
- Studies which reported magnitude of depression in PLWHIV
- Articles that assessed risk factors of depression in PLWHIV
- Studies done in east Africa

### Exclusion criteria

- We excluded commentaries, editorials, letters, reviews, and interventional studies
- Duplicate studies were also excluded

### Methods for data extraction and quality assessment

We used standardized data extraction form to extract data from identified studies. The following information were extracted for each included study: The name of the first author, publication date, study design, associated factors, sample size study setting, tools used for assessing outcome, confounders adjusted for, risk estimate (OR) and their 95% confidence interval. Data extraction from source documents was done independently by two investigators. Disagreements were resolved by consensus.

The quality of included studies was evaluated by using a modified version of the Newcastle-Ottawa Scale (NOS) [33]. Sample representativeness and size, comparability between participants, ascertainment of depressive symptoms, and statistical quality were the domains NOS scale uses to assess the quality of each studies. Actual agreement and agreement beyond chance (unweighted Kappa) were used to evaluate the agreement between the two reviewers. We considered the values 0 as poor agreement, 0.01–0.20 as slight agreement, 0.21– 0.40 as fair agreement, 0.41–0.60 = moderate agreement, 0.61– 0.80 as substantial agreement, and 0.81–1.00 as almost perfect agreement [34].

### Data synthesis and analysis

Comprehensive meta-analysis software version3 was used for meta-analysis and forest plots that showed combined estimates with 95%CI. The overall pooled prevalence was estimated by random effect meta-analysis [35]. Heterogeneity was evaluated using Q statistic and the I^2^statistics [35]. The magnitude of statistical heterogeneity between studies was assessed using *I*^2^ statistic and values of 25, 50 and 75% were considered to represent low, medium and high, respectively [36]**.** For the data identified as heterogeneous, a random-effects model was used during analysis. When statistical pooling is not possible, non-pooled data was presented in table form. Meta-regression was performed to explore the potential source of heterogeneity.

The instrument used to assess depression and the country where the studies were conducted were used to determine the possible source of heterogeneity between the studies. In addition, we carried out a leave-one-out sensitivity analysis to evaluate the key studies that exert major impact on between-study heterogeneity. Publication bias was assessed by funnel plot and Egger’s regression tests

## Results

### Identification of studies

**Fig. 1:**
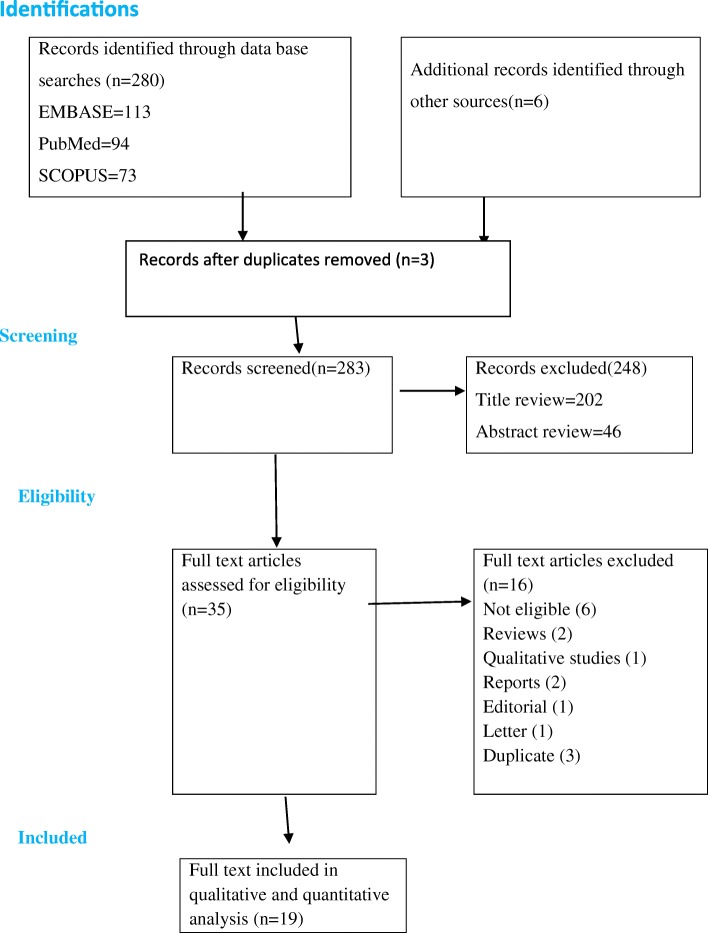
PRISMA flowchart of review search

We identified 280 articles in our database search. Our manual search resulted additional six articles. Of these we excluded 205 articles during the review of duplicate and titles as they did not met the inclusion criteria (Fig. 1). In our review of abstracts and keywords we excluded another 46 studies and 19 articles with full-text that met the inclusion criteria were included in our final analysis.

### Characteristics of included studies

Among the total of nineteen studies included in our analysis ten of them were done in Uganda [5, 7, 8, 10, 11, 20, 26, 37–39], whereas five of the studies were conducted in Ethiopia [2, 3, 9, 16, 17] and the other studies were conducted in Kenya [18], Tanzania [15], Rwanda [14] and Sudan [40]. The publication year of those studies ranged from 2006 to 2017. All of the included studies used cross-sectional study design. One study used community samples [11] and eighteen studies used samples from institution [2, 3, 5, 7–10, 14–18, 20, 26, 37–40]. Regarding the instrument used, four studies used diagnostic instrument [5, 10, 15, 38], fourteen studies used screening instrument [2, 3, 7–9, 14, 16, 17, 26, 37, 39, 40] and one study used self-report questionnaire to assess depression in PLWHIV [11]. Majority(84.22%) of the studies not reported response rate. Seven studies used random sampling technique, seven used the cohort sample, one study used covenant sampling and four studies not reported the sampling technique (See Table 1).

**Table 1:** Distribution of studies on depression in people living with HIV included in qualitative and quantitative analysis based on year, study design, sample size, instrument, country, response rate, study population and prevalence

Author (year) (reference number)	Study design(setting)	Sample size	Tool	Response rate	Country	Sampling	Study population	Outcome (magnitude of depression)
Psaros C. et. al. (2014) [37]	Cross sectional study (institution based)	453	HSC-D	Not reported	Uganda	Used Cohort population sample	adults	38%(*n*=172)
Shumba C,et. al. (2013) [11]	Cross sectional study (Community based study)	732	Self report	Not reported	Uganda	Random sampling (specific technique not reported)	Adults on HAART	59%(*n*=429)
Kinyanda E. et. al. (2017) [5]	Cross sectional study (institution based study)	899	DSM	Not reported	Uganda	Not reported	adults	14%(*n*=126)
Yeneabat T.et. al. (2017) [9]	Cross sectional study (institution)	390	CES-D	Not reported	Ethiopia	Not reported	adults	76.7%(*n*=299)
Kinyanda E. et. al. (2011) [38]	Cross sectional study (institution)	618	DSM-IV	Not reported	Uganda	Not reported	adults	8.1%(*n*=50)
Nakasujja N. et. al. (2010) [39]	Cross sectional study (institution setting)	102	CES-D	Cohort survey	Uganda	Not reported	Adults	53.9%(*n*=55)
Akena D. et. al. (2012) [26]	Cross sectional study (institution setting)	368	PHQ-9	Not reported	Uganda	Simple random sampling	Adults	17.4%(*n*=64)
Hatcher AM. et. al. (2012) [7]	Cross sectional study (institution setting)	270	HSC-D	Not reported	Uganda	Used Cohort population sample	Adults	23.7%(*n*=64)
Cohen MH.et. al. (2009) [14]	Cross sectional study (institution setting)	658	CES-D	Not reported	Rwanda	Used Cohort population sample	Adults	81%(*n*=533)
Musisi S. et. al. (2014) [8]	Cross sectional study (institution setting)	386	PHQ-9	Not reported	Uganda	Used cohort population sample	Adults	30%(n=116)
Tesfaw G.et. al. (2016) [2]	Cross sectional study (institution setting)	417	HADS	100%	Ethiopia	Systematic random sampling	Adults	41.2%(*n*=172)
Elbadawi A.et. al. (2017) [40]	Cross sectional study (institution setting)	362	HADS	Not reported	Sudan	Systematic random sampling	Adults	61.3%(*n*=222)
Eshetu DA. et. al. (2014) [17]	Cross sectional study (institution setting)	416	PHQ-9	Not reported	Ethiopia	Systematic random sampling	Adults	38.94%(*n*=162)
Berhe H. et. al. (2013) [3]	Cross sectional study (institution setting)	269	HAM-D	Not reported	Ethiopia	Not reported	adults	43.9%(*n*=118)
Mohammed M. et. al. (2015) [15]	Cross sectional study (institution setting)	740	PHQ-9	97%	Ethiopia	Systematic random sampling	Adults	45.8%(*n*=339)
MB.ChB.PW (2011) [18]	Cross sectional study (institution setting)	400	BDI	Not reported	Kenya	Systematic random sampling		47.25%(*n*=189)
Marwick KF. et. al. (2010) [15]	Cross sectional study (institution setting)	220	ICD-10	97%	Tanzania	Convenience sampling	Adults	2.7%(*n*=6)
Kahazura FM. et. al. (2006) [20]	Cross sectional study (institution setting)	1017	CES-D	Not reported	Uganda	Used Cohort population sample	Adults	47%(*n*=476)
Mpungu EN.et. al, 2011 [10]	Cross sectional study (institution setting)	500	DSM (MINI)	Not reported	Ugnada	Not clearly indicated	adults	46.4%(232)

Key: *DSM* Diagnostic and Statistical Manual of Mental Disorders, *ICD* International Classification of Disease, *PHQ-9* Patient Health Questionnaire-9, *CES-D* Center for Epidemiologic Studies Depression Scale Revised, *HAM-D* Hamilton Depression Rating Scale, *BDI* Beck's Depression Inventory, *HADS* Hospital Anxiety and Depression Scale, *HSC-D* Hopkins Symptom Checklist for Depression

### Quality of included studies

We used a modified version of the Newcastle-Ottawa scale (NOS) scale to evaluate the quality of included studies. The methodological quality was good for all nineteen studies. The risk of selection, measurement and non-response bias was low for all studies. The agreement between reviewers regarding the level of bias was moderate or almost perfect for all studies (Kappa statistic range 0.50-1 (Additional file 1: Table S1).

### The results of pooled meta-analysis

#### Prevalence of depression in people living with HIV (PLWHIV)

**Fig. 2:**
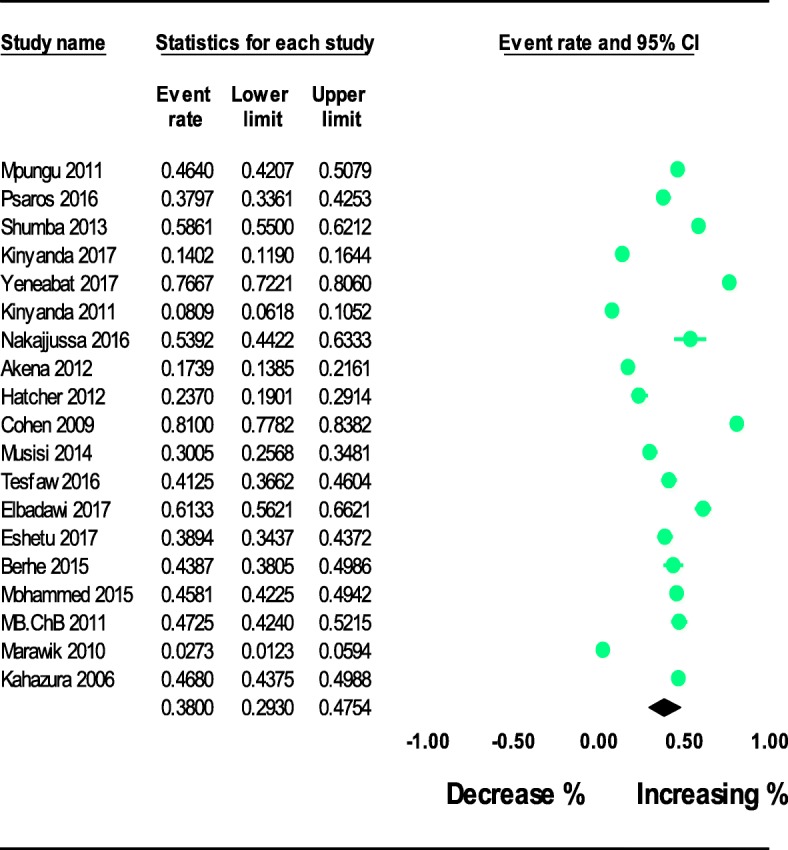
The prevalence of depression in people living with HIV in East Africa: meta-analysis

Nineteen of the studies provided information regarding the prevalence of depression in PLWHIV in east Africa [1–19] (Table 1). These nineteen studies could be combined to provide the pooled estimates. Based on the results of random-effects method, the pooled prevalence of depression in PLWHIV was 38.00% (95% CI 29.30-47.54) and the heterogeneity was considerable (*I*^2^=98.59%; Q=11276.10, df=18, variance= 0.08, *p* <0.0001). The forest plots of the prevalence of depression in people living with HIV were shown in Fig. 2.

#### Subgroup analyses of the prevalence of depression in people living with HIV (PLWHIV)

Subgroup analysis were performed by type of instrument used to assess depression and the country where the studies were conducted as possible source of heterogeneity between the studies as well as to explore the prevalence in each country. For our subgroup analysis based on the country where the studies were conducted, we identified representative data only for Uganda and Ethiopia. For the rest of east African countries (Kenya, Tanzania, Rwanda and Sudan), there is limited data for subgroup analysis.

Of the included studies, ten reported the prevalence of depression in PLWHIV in Uganda [1–4, 6–9, 11, 19, 20]. The pooled prevalence estimates of depression in PLWHIV in Ugandawas30.88% (95% CI 20.84-43.13) (See Table 2).

**Table 2:** Prevalence of depression in people with HIV in east Africa: Subgroup meta-analysis and heterogeneity analysis

Characteristics	Observation (N)	Prevalence (%)	95% CI	I2	Q	df	*P* value
Geographic location or country							
Uganda	10	30.88	20.84-43.13	98.57%	636.65	9	*P*<0.0001
Ethiopia	5	49.79	37.22-62.38	97.08	136.79	4	*P*<0.0001
Instrument used or tool used							
Diagnostic	4	12.40	4.0-32.80	98.94%	281.77	3	*P*<0.0001
Screening	14	46.00	36.90-55.38	97.96%	638.36	13	*P*<0.0001
Study Setting							
Institution based	18	36.86	27.87-46.86	98.59%	1207.91	17	*P*<0.0001
Sample size							
<400	8	34.20	19.84-52.18	98.32%	416.89	7	*P*<0.0001
>400	11	40.46	29.39-52.60	98.84%	858.66	10	*P*<0.0001
Reference to diagnostic criteria							
DSM/ICD	4	12.40	4.0-32.80	98.94%	281.77	3	*P*<0.0001
PHQ-9	4	32.15	21.58-44.92	96.61%	88.57		*P*<0.0001
HADS	2	51.30	32.20-70.10	96.75%	30.82	1	*P*<0.0001
CES-D	4	66.14	44.34-82.73	98.67%	226.22	3	*P*<0.0001
HSC-D	2	30.50	18.50-46.10	93.51%	14.41	1	*P*<0.0001
Year of publication							
2006-2010	3	33.90	10.17-69.93	99.25%	267.34	2	*P*<0.0001
2011-2013	7	32.03	19.49-47.84	98.52%	406.32	6	*P*<0.0001
2014-2017	9	43.50	31.23-56.69	98.37%	98.37% 489.63	8	*P*<0.0001

Five studies reported the prevalence of depression in PLWHIV in Ethiopia [5, 12, 14–16]. The pooled prevalence of depression in PLWHIV in Ethiopia was 49.79% (95% CI37.22-62.38**)**. (See Table 2). In our analysis the prevalence of depression in PLWHIV in Ethiopia is significantly higher than the prevalent in Uganda (*P*<.00001). For the rest of east African countries, there is limited data for subgroup analysis. We found only one study conducted in each of the other four east African countries such as Kenya [18], Tanzania [15], Rwanda [14] and Sudan [40].

We also performed subgroup analysis based on the instrument used to assess depression. Four studies used diagnostic instrument (DSM or ICD) and [1, 4, 6, 18, 20] and fourteen studies used screening instrument (PHQ-9, HADS, BDI, CES-D, or HSC-D) to assess depression in people living with HIV in east Africa [1, 5, 7–17, 19]. The pooled prevalence of depression in people living with HIV was 12.40% (95% CI 4.0-32.80) and 46% (95% CI 36.90-55.38**)** for the studies conducted using diagnostic and screening instrument respectively (see Table 2).

Furthermore, we conducted subgroup analysis based on each instrument used to assess depression. The pooled prevalence estimates of depression for the studies conducted using CES-D 66.14% (44.34-82.73) and HADS 51.30% (32.20-70.10) were significantly higher than results of studies conducted using DSM or ICD12.40% (95% CI 4.0-32.80), PHQ-9 32.15% (21.58-44.92), and HSC-D 30.50% (18.50-46.10) (see Table 2).

Finally, we performed subgroup analysis based on the setting where the studies were conducted. The pooled prevalence of depression in people living with HIV in east Africa for institution based studies was 36.86% (95% CI 27.87-46.86) (see Table 2).

#### Publication bias

**Fig 3:**
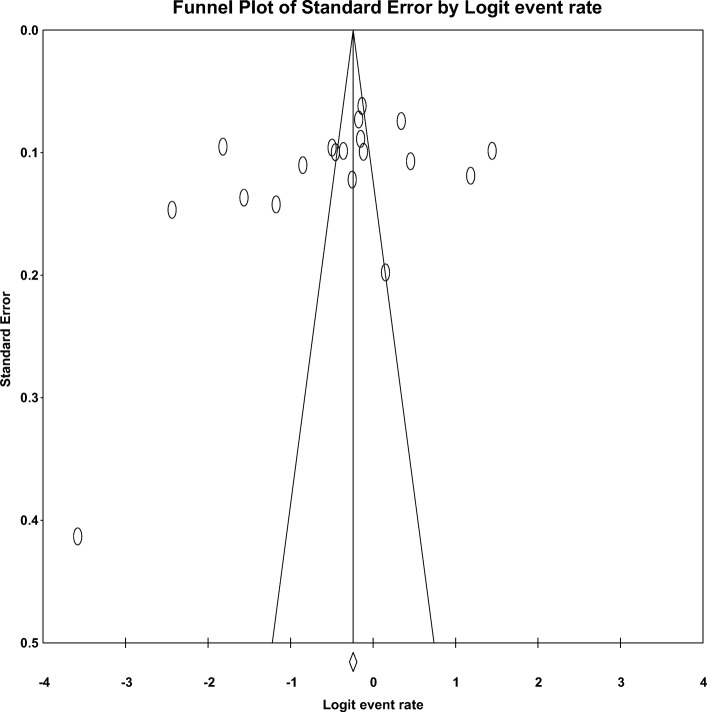
Funnel plot of risk of publication bias for the prevalence of depression in PLWHIV in east Africa

The funnel plot and Egger’s regression tests (B=-9.17, SE=6.16, P=0.155) provided no evidence of substantial publication bias for the prevalence of depression in PLWHIV in east Africa (Fig. 3).

#### Sensitivity analysis

For the purpose of further investigating potential source of heterogeneity in the analysis of the prevalence of depression in PLWHIV, we performed leave-one-out sensitivity analysis. Our sensitivity analysis showed that our findings were strong and not dependent on a single study. Our pooled estimated prevalence varied between 35.86(27.53-44.25) and 41.39(32.33-51.07) after deletion of a single study (see Table 3).

**Table 3:** Sensitivity analysis of prevalence for each study being removed at a time: prevalence and 95% confidence interval of depression in people living with HIV in east Africa

Study excluded	Prevalence	95%CI
Mpungu 2011	37.49	28.32–47.66
Psaros 2016	37.96	28.79–48.08
Shumba 2013	36.86	27.87–46.86
Kinyanda 2017	39.90	31.51–48.94
Yeneabat 2017	35.86	27.53–45.14
Kinyanda 2011	40.67	32.13–49.82
Nakajjussa 2016	37.17	28.31–46.99
Akena 2012	39.44	30.52–49.11
Hatcher 2012	38.89	29.88–48.74
Cohen 2009	35.59	27.78–44.25
Musisi 2014	38.45	29.67–48.42
Tesfaw 2016	37.78	28.63–47.89
Elbadawi 2017	36.74	27.90–46.56
Eshetu 2017	37.91	28.76–48.01
Berhe 2015	37.65	28.60–47.65
Mohammed 2015	37.50	28.19–47.84
MB.ChB 2011	37.46	28.36–47.54
Marawik 2010	41.39	32.33–51.07
Kahazura 2006	37.43	27.98–47.95

Key. The analysis is based on random effect model

#### Determinants of depression in people living with HIV

Of 19 included studies, 11 studies that reported factors associated with depression in peoples living with HIV in east Africa were included in the qualitative analysis [2, 3, 5, 7, 9, 10, 16, 17, 20, 26, 38] (See Table 4). Association between WHO clinical staging of disease and depression was observed in three studies [2, 10, 17].

**Table 4:** Characteristics of factors associated with depression in people living with HIV east Africa by their odds ratio, confidence interval strength of association, author and year

Factors	Odds ratio (AOR)	95% Confidence interval	Strength of association	Adjusted for	Author, year
Past history of mania	2.73	1.16–6.40	Moderate, negative	Gender, age, educational status, income, social support, self-efficacy, cognitive impairment, current alcohol use, current alcohol use disorder, duration of ART, CD4 count, WHO clinical staging of HIV disease, tuberculosis	Mpungu, 2011
Mean self efficacy score > 89%	0.61	0.41–0.90	Strong, negative	Gender, age, educational status, income, social support, mania, cognitive impairment, current alcohol use, current alcohol use disorder, duration of ART, CD4 count, WHO clinical staging of HIV disease, tuberculosis	Mpungu, 2011
WHO clinical staging of HIV disease	1.88	1.24–2.44	Moderate, positive	Gender, age, educational status, income, social support, mania, self-efficacy, cognitive impairment, current alcohol use, current alcohol use disorder, duration of ART, CD4 count, mania, tuberculosis	Mpungu, 2011
Tuberculosis	2.37	1.20–4.66	Moderate, positive	Gender, age, educational status, income, social support, mania, self-efficacy, cognitive impairment, current alcohol use, current alcohol use disorder, duration of ART, CD4 count, mania, WHO clinical staging of HIV disease	Mpungu, 2011
Age group 40–49	0.95	0.92–0.98	Strong, negative	AIDS stigma, CD4 counts, sex	Akena, 2012
AIDS stigma	1.65	1.20–1.26	Moderate, positive	Age, CD4 counts, sex	Akena, 2012
CD4 count	0.43	0.20–0.91	Moderate, negative	Age, AIDS stigma, sex	Akena, 2012
Not have enough food security	2.89	1.40–5.98	Moderate, positive	Distance from HIV clinic, When knew HIV status, On ART, social support, negative life events, stress score index	Kinyanda. 2011
Negative life event score 6–10	4.89	1.83–10.06	Strong, positive	Distance from HIV clinic, When knew HIV status, On ART, social support, food security, stress score index	Kinyanda. 2011
Negative life event score 6–11+	16.67	7.01–39.66	Strong, positive	Distance from HIV clinic, When knew HIV status, On ART, social support, food security, stress score index	Kinyanda. 2011
Stress score index> 10	7.18	2.65–19.47	Strong, positive	Distance from HIV clinic, When knew HIV status, On ART, social support, food security, negative life event	Kinyanda. 2011
Food insecurity	3.83	1.58–9.32	Strong, positive	Sex, age educational status, marital status, occupation, residence, number of dependent children, access to food aids, practice of agriculture, ownership of livestock, CD4 level, OIs	Yeneabat, 2017
Presence of OIs	5.20	1.34–20.16	Strong, negative	Sex, age educational status, marital status, occupation, residence, number of dependent children, access to food aids, practice of agriculture, ownership of livestock, CD4 level, food insecurity	Yeneabat, 2017
HIV stage three	2.80	1.50–5.21	Moderate, positive	Age, educational status, marital status, residence, CD4 level, perceived stigma, medication adherence, social support	Tesfaw, 2016
Perceived stigma	3.60	2.23–5.80	Strong, positive	Age, educational status, marital status, residence, CD4 level, HIV stage, medication adherence, socia; support	Tesfaw, 2016
Medication adherence	1.61	1.02–2.55	Moderate, positive	Age, educational status, marital status, residence, CD4 level, HIV stage, social support	Tesfaw, 2016
Social support	2.02	1.25–3.27	Moderate, positive	Age, educational status, marital status, residence, CD4 level, HIV stage, medication adherence	Tesfaw, 2016
Married	0.01	0.01–0.07	Weak, positive	Sexual relationship power scale, age, educational status, heavy drinking, tobacco use, WHO HIV stage, CD4 count, previously treated with ART	Hatcher, 2012
Resilience score	1.28	1.12–1.45	Weak, positive	Study site, age, sex, stigma score, childhood trauma score, negative coping score, negative life event experience	Kinyanda, 2017
Stigma score	1.37	1.08–1.73	Weak, positive	Study site, age, sex, resilience childhood trauma score, negative coping score, negative life event experience	Kinyanda, 2017
Childhood trauma score	1.25	1.02–1.55	Weak, positive	Study site, age, sex, resilience stigma score, negative coping score, negative life event experience	Kinyanda, 2017
Negative scoping score	1.50	1.17–1.92	Moderate, positive	Study site, age, sex, resilience stigma score, childhood trauma, negative life event experience	Kinyanda, 2017
Negative life experience score	1.81	1.46–2.25	Moderate, positive	Study site, age, sex, resilience stigma score, childhood trauma, *negative scoring score*	Kinyanda, 2017
Male sex	1.633	1.138–2.342	Moderate, positive	Marital status,income, verbal stigma, missed frequency of clinic visit, frequency of clinic visit/month	Mohammed, 2016
Monthly income<500eth.birr	1.924	1.159,3.195	Moderate, positive	Marital status, sex,, verbal stigma, missed frequency of clinic visit, frequency of clinic visit/month	Mohammed, 2016
Had verbal stigma	2.705	1.445,5.063	Moderate, positive	Marital status, sex,, verbal stigma, missed frequency of clinic visit, frequency of clinic visit/month	Mohammed, 2016
Missed frequency of clinic visit past one,two and three weeks	4.35 4.56 and 3.75 respectively	2.20–8.59, 2.42–8.58 and 1.39–9.93 respectively	Strong, positive	Marital status, sex,, verbal stigma, frequency of clinic visit/month	Mohammed, 2016
Once, twice and three times frequency of clinic visit/month	19.033, 13.784 and 22.729	2.095–172.878, 1.430–132.871, and 2.450–210.873 respectively	Strong, positive	Marital status, sex,, verbal stigma, verbal stigma, missed frequency of clinic visit	Mohammed, 2016
Female sex	2.071	1.077–3.985	Moderate, positive	Age, income, HIV stage, hospitalization in past one month, felt stigmatized	Eshetu 2015
Age 30–39	2.761	1.165–6.540	Moderate, positive	Sex, Income, HIV stage, hospitalization in past one month, felt stigmatized	Eshetu 2015
Age 50–59	2.596	1.49–9.94	Moderate, positive	Sex, Income, HIV stage, hospitalization in past one month, felt stigmatized	Eshetu 2015
Age 60–69	19.645	4.02–95.99	Strong, positive	Sex, Income, HIV stage, hospitalization in past one month, felt stigmatized	Eshetu 2015
Income < 200 Birr	3.917	1.559, 9.845	Strong, positive	Age,Sex, HIV stage, hospitalization in past one month, felt stigmatized	Eshetu 2015
Income201–400 Birr	2.796	1.139–6.865	Moderate, positive	Age, Sex, HIV stage, hospitalization in past one month, felt stigmatized	Eshetu 2015
Income 401–700 Birr	2.590	1.058–6.340	Moderate, positive	Age, Sex, HIV stage, hospitalization in past one month, felt stigmatized	Eshetu 2015
HIVStage III	2.317	1.108–4.85	Moderate, positive	Age, Sex, income hospitalization in past one month, felt stigmatized	Eshetu 2015
HIV Stage Iv	8.769	1.93–39.87	Strong, positive	Age, Sex, income hospitalization in past one month, felt stigmatized	Eshetu 2015
Hospitalized in the past one Month	15.26	1.463–159.22	Strong, positive	Age, Sex, income month, felt stigmatized, HIVStage	Eshetu 2015
Felt stigmatized	3.597	1.86–6.95	Strong, positive	Age, Sex, income month,, HIVStage, hospitalized in the past one Month	Eshetu 2015
Age > 50 years	1.93	1.09–3.42	Moderate, positive	Religion, education, marital status, sex, source of income, CD4 level, HIVStage, number of living children	Kaharuza, 2006
Pensions as source of income	1.81	1.24–2.66	Moderate, positive	Religion, education, marital status, sex, CD4 level, HIVStage, number of living children, age	Kaharuza, 2006
Primary education	1.69	1.12–2.52	Moderate, positive	Religion, marital status, sex, CD4 level, HIVStage, number of living children, age, source of income	Kaharuza, 2006
CD4 count 50–99	2.02	1.22–3.36	Moderate, positive	Religion, marital status, sex, education, HIVStage, number of living children, age, source of income	Kaharuza, 2006
CD4 count < 50	2.34	1.39–3.93	Moderate, positive	Religion, education, marital status, sex, HIVStage, number of living children, age, source of income	Kaharuza, 2006
Urban residence	3.19	1.50–6.65	Strong, positive	Sex, marital status, income, educational status, occupation, CD4 level	Berhe, 2013
Income < 200 ETB	4.43	1.35–14.58	Strong, positive	Sex, residence, marital status,, educational status, occupation, CD4 level	Berhe, 2013
Unemployed	2.74	1.34–5.57	Moderate, positive	Sex, residence, marital status, educational status, CD4 level	Berhe, 2013
Government employee	3.56	1.73–7.30	Strong, positive	Sex, residence, marital status, educational status, CD4 level	Berhe, 2013

In the current review presence of opportunistic infection [9], perceived stigma [2, 20], negative life event [38], WHO clinical staging of disease [17], hospitalization in the past one month [17], stressful life events [38], food insecurity [9], mean self-efficacy (>80%) [41], missed frequency of clinic visit one to three weeks [16], frequency of follow-up [16], older age (age 60-69) [17], income less than 200 Ethiopian birr [3, 17], urban residence [3] and government employee [3] were strongly and significantly associated with depression in people living with HIV in east Africa (See Table 4).

## Discussion

To our knowledge, this is the first systematic review and meta-analysis of depression and determinants in PLWHIV in east Africa. The main aim of the study was identification of epidemiological information on the overall prevalence and common determinants of depression in PLWHIV in the area. It is also believed that the results of this review provide greater precision than the results of the studies individually considered. Nineteen studies that analyzed the prevalence of depression and associated factors in PLWHIV in east Africa were included.

The pooled prevalence estimate of depression in this meta-analysis was found a to be 38% in PLWHIV. The magnitude of depression in PLWHIV differed by the setting and the country where the studies were conducted as well as the type of instrument used to measure depression. When measured by DSM or ICD the estimated pooled prevalence of major depressive disorder appeared to be 12.40 % , but the pooled prevalence of depression was 46% as measured by screening instruments. The magnitude of major depressive disorder in PLWHIV in our meta-analysis is much higher compared with the 4% found in the general population [42].

Our estimated pooled prevalence of depression (38%) was higher than the results of systematic review and meta-analysis studies in sub-Saharan Africa which reported the pooled prevalence estimates of depression was 19% in people living HIV with sub-Saharan Africa [22]. These difference might be due to the socioeconomic and cultural differences of the countries.

In our study the pooled prevalence estimates of depression in PLWHIV in Ethiopia (49.79%) was significantly higher than the pooled prevalence estimates of depression in Uganda(30.88%). The difference might be due to all studies done in Ethiopia used screening instrument to determine depression and based on the pooled prevalence estimate, the prevalence of depression measured by screening instrument(46%) was higher than the prevalence of depression measured by diagnostic instrument (12.40%).

As expected, the magnitude of depression showed a considerable variations depending on the measurement used to determine depression in PLWHIV. The pooled prevalence of depression in PLWHIV was apparently higher in studies conducted using screening instrument than diagnostic instrument. The estimated pooled prevalence of depression in PLWHIV was found to be 66.14% , 51.30%, 12.40%, 32.15% and 30.50% as measured by CES-D, HADS, DSM or ICD, PHQ-9, and HSC-D respectively. The fact that diagnostic instrument give more weights towards the high specificity as compared to screening instrument which gives more emphasis towards high sensitivity might be the main reason for the observed difference. The other possible explanation might be diagnostic instrument uses strict criteria as compared to screening instrument as screening instrument aimed to identify potential indicators for disease or potential disease rather than establishing the presence or absence of disease which is the main purpose of diagnostic instrument. Finally, the possible interpretation for the variation in magnitude of depression among the different screening instrument may be due to difference in the sensitivity and specificity the tools used to screen depression in PLWHIV. The findings support the view that validation and use of standard instrument for screening as well as diagnosis of depression in PLWHIV.

Moreover, in our subgroup analysis of prevalence in PLWHIV by study setting show the pooled prevalence of depression in PLWHIV for the studies conducted institution-based setting was 36.86%. This results are lower than unpooled results of depression in community setting which reported magnitude of depression 59%. (see Tables 1 and 2). The fact that the community based study included in this review used self report of depression and all institution based studies used standard diagnostic or screening instrument to assess depression might be the possible reason for the observed difference in magnitude of depression in clinical and community based settings. In addition, the community based study is only one study so the observed result might be highly biased as it is self-report. The study suggest replication of studies in community samples to strengthen our finding as well as understanding of the precise estimates of depression in PLWHIV.

Concerning associated factors, Our qualitative synthesis showed that factors such as having opportunistic infection, perceived stigma, negative life event, WHO clinical staging of disease, hospitalization in the past one month, stressful life events, food insecurity, self-efficacy, missed frequency of clinic visit, frequency of follow-up, older age, low income, urban residence and being government employee were strongly and significantly associated with depression in people living with HIV in east Africa.

### Difference between studies

The variation between the studies included in our systematic review and meta-analysis led to high level of heterogeneity in our analysis. The possible contributing factors for the variance in prevalence rates of depression in PLWHIV in east Africa includes the instrument used to measure depression, the study setting and populations. We conducted leave-one-out sensitivity analysis for the purpose of further investigating potential source of heterogeneity in the analysis of the prevalence of depression in PLWHIV. Our sensitivity analysis showed that our findings were strong and not dependent on a single study.

### Strength and limitations

The strength of the study includes the use of predefined search strategy in order to reduce reviewers bias as well as performing data extraction and quality assessment by two independent reviewers to minimize the possible reviewer bias. The other strength is performing sensitivity and subgroup analysis based on study setting, instrument used, and country of study to identify the small study effect and the risk of heterogeneity. Finally, evaluating the quality of the studies included in analysis and based on the results from the assessment of the study quality the methodological quality was generally good. Nevertheless, the study has some limitations: first, small number of studies were included in our subgroup analysis which reduce the precision of the estimate; second, apparent heterogeneity was identified among the studies; due to inconsistent adjustment and inclusion of factors determining depression in PLWHIV we done only qualitative analysis for associated factors of depression.

## Conclusion

Our study found that the pooled prevalence estimate of depression in PLWHIV was 38% (95% CI 29.30-47.54). In our study the pooled prevalence estimates of depression in PLWHIV in Ethiopia was significantly higher than the pooled prevalence estimates of depression in Uganda. In addition the prevalence of depression was significantly higher in studies conducted by using screening as compared to diagnostic instrument. The study also resulted in a significant variation in prevalence of depression based on the instrument used. These findings have several implications for clinical practice and research. Based on this finding routine screening and integrated management of mental health condition into the existing HIV care services is warranted in east Africa. Validation and use of standard instrument to assess depression in PLWHIV is needed. In addition longitudinal and community based studies focusing on incidence and determinates of depression in PLWHIV are recommended. Attention need to be given for those people who have opportunistic infection, older age, advanced HIV stage, experienced stressful life events,who have perceived stigma and those who are on ART to prevent risk of non-adherence and treatment resistance and further to improve the quality PLWHIV.

## Additional files

Additional file 1: **Table S1.** Summary of agreed level of bias and level of agreement on the methodological qualities of included studies in meta-analysis based on sampling, outcome, response rate and method of analysis. (DOCX 15 kb)

## Abbreviations

BDI: Beck's Depression Inventory
CES-D: Center for Epidemiologic Studies Depression Scale Revised
DSM: Diagnostic and Statistical Manual of Mental Disorders
HADS: Hospital Anxiety and Depression Scale
HAM-D: Hamilton Depression Rating Scale
HIV: Human Immunodeficiency Virus
HSC-D: Hopkins Symptom Checklist for Depression
ICD: International Classification of Disease
PHQ-9: Patient Health Questionnaire-9,
PLWHIV: people living with Human Immunodeficiency Virus
