# Supplementary material for: A systematic review and meta-analysis of epidemiology of depression in people living with HIV in east Africa
Source: BMC Psychiatry. 2018 Aug 15;18:254. doi: 10.1186/s12888-018-1835-3 (PMC6094569; doi:10.1186/s12888-018-1835-3)
Supplement: Supplementary file 1 — Table S1. Summary of agreed level of bias and level of agreement on the methodological qualities of included studies in meta-analysis based on sampling, outcome, response rate and method of analysis. (DOCX 15 kb) [file 12888_2018_1835_MOESM1_ESM.docx]

**Table S1** Summary of agreed level of bias and level of agreement on the methodological qualities of included studies in meta-analysis based on sampling, outcome, response rate and method of analysis

| Study | Overall agreement and precision | | |
| --- | --- | --- | --- |
|  | Percentage of agreement | Kappa value | Level of agreement |
| Mpungu 2011 | 75 | 0.60 | Moderate |
| Psaros 2016 | 100 | 1 | Almost perfect |
| Shumba 2013 | 75 | 0.50 | Moderate |
| Kinyanda 2017 | 75 | 0.60 | Moderate |
| Yeneabat 2017 | 75 | 0.60 | Moderate |
| Kinyanda 2011 | 75 | 0.60 | Moderate |
| Nakajjussa 2016 | 100 | 1 | Almost perfect |
| Akena 2012 | 100 | 1 | Almost perfect |
| Hatcher 2012 | 100 | 1 | Almost perfect |
| Cohen 2009 | 100 | 1 | Almost perfect |
| Musisi 2014 | 100 | 1 | Almost perfect |
| Tesfaw 2016 | 100 | 1 | Almost perfect |
| Elbadawi 2017 | 100 | 1 | Almost perfect |
| Eshetu 2017 | 100 | 1 | Almost perfect |
| Berhe 2015 | 100 | 1 | Almost perfect |
| Mohammed 2015 | 100 | 1 | Almost perfect |
| MB.ChB 2011 | 75 | 0.60 | Moderate |
| Marawik 2010 | 75 | 0.50 | Moderate |
| Kahazura 2006 | 100 | 1 | Almost perfect |
